# 

**Published:** 1894-10-27

**Authors:** 


					No. 422. Vol. XVII.
"aturday,
Oct. 27th, 1894.
WITH EXTRA NUR8ING SUPPLEMENT. price twopence.
[Registered at the General Post Office as a Newspaper for P0!1/?0-
transmission in the United Kingdom and Abroad.] Ditto^rKfil.CiS?&

				

